# Supplementary material for: FineST: contrastive learning integrates histology and spatial transcriptomics for nuclei-resolved ligand-receptor analysis
Source: Nat Commun. 2026 Mar 16;17:4645. doi: 10.1038/s41467-026-70528-7 (PMC13201544; doi:10.1038/s41467-026-70528-7)
Supplement: Supplementary file 1 — Supplementary Information [file 41467_2026_70528_MOESM1_ESM.pdf]

Supplementary Information for

FineST: Contrastive learning integrates histology and spatial transcriptomics for nuclei-resolved ligand-receptor analysis

Lingyu Li[1], Tianjie Wang[1], Zhuo Liang[2], Huajian Yu[1], Stephanie Ma[1,3], Lequan Yu[2,*], Yuanhua Huang[1,2,4,*]

[1]School of Biomedical Sciences, The University of Hong Kong, Hong Kong SAR, China
[2]School of Computing and Data Science, The University of Hong Kong, Hong Kong SAR, China
[3]State Key Laboratory of Liver Research, The University of Hong Kong, Hong Kong SAR, China
[4]InnoHK-Centre for Translational Stem Cell Biology, Hong Kong Science Park, Hong Kong SAR, China

*Corresponding author: lqyu@hku.hk, yuanhua@hku.hk

Contents

S1 Supplementary Methods **2**
- S1.1 Extraction of ligand-receptor gene expression profiles . . . 2
- S1.2 Extraction of highly variable gene expression profiles . . . 2
- S1.3 Spots interpolating . . . 2
- S1.4 Computational constraints for image feature extraction . . . 3
- S1.5 Symbolic representation in Moran's R . . . 3
- S1.6 Visualization of sender and receiver cell types for LRIs . . . 4

S2 Supplementary Tables **5**
- Table S1. The datasets analyzed in FineST. . . . 5
- Table S2. Sparsity in representative VisiumHD datasets. . . . 6
- Table S3. The supporting of FineST's identified two key LR pairs in previous studies. . . . 6
- Table S4. The comparison of FineST and iStar that run with the same setup. . . . 7
- Table S5. Top 20 features of SPPI+ macrophage and CAF from literature [1]. . . . 7
- Table S6. The pre-trained Vision Transformer (ViT) used in FineST. . . . 7
- Table S7. Significant LR pairs detected in Visium HCC ROI. . . . 7
- Table S8. The software that compares with FineST. . . . 8

S3 Supplementary Figures **9**
- Figure S1. The overview of FineST (Fine-grained Spatial Transcriptomics). . . . 9
- Figure S2. The imputation and downstream tasks of FineST. . . . 10
- Figure S3. FineST achieved accurate prediction and pinpointed cell types on the VisiumHD CRC dataset with 16μm resolution. . . . 11
- Figure S4. FineST facilitated more refined cell clusters on the VisiumHD CRC dataset with 16μm resolution. . . . 12
- Figure S5. FineST discovered intricate ligand-receptor pairs and cell-cell communication patterns on CRC dataset with 16μm resolution. . . . 13
- Figure S6. FineST outperforms iStar in gene expression prediction and identifies biologically relevant ligand-receptor pairs at the single-cell level. . . . 14
- Figure S7. FineST detected fine-grained ligand-receptor interaction on the Visium NPC dataset. . . . 15
- Figure S8. Super-resolved ST expression profile evaluation on the Visium NPC dataset. . . . 16
- Figure S9. FineST-identified LR pairs are biologically meaningful and mechanistically distinct, highlighting the value of high-resolution CCC analysis. . . . 17
- Figure S10. FineST achieved region-specific fine-grained ligand-receptor interaction within selected ROIs. 18
- Figure S11. FineST discovered fine-grained LR interaction at the boundary of Tregs and Tumor cells. 19
- Figure S12. FineST outperforms iStar in gene expression prediction and uncovers putative cellular crosstalk within tumor-immune barrier in HCC. . . . 20
- Figure S13. Comparison of inference performance between iStar and FineST methods. . . . 21
- Figure S14. The HE image segmentation for sub-spot and single-nuclei in FineST framework. . . . 22

S1 Supplementary Methods

S1.1 Extraction of ligand-receptor gene expression profiles

Let the spatial gene expression matrix be $Y \in \mathbb{N}^{n\times p}$ with the spot coordinate representation $S \in \mathbb{R}^{n\times 2}$, where n is the number of spatial spot in-tissue of given section, p be the number of genes, and $S = (s_1, s_2, \cdots, s_n)^\top$ with $s_k = (x_k, y_k)$ denoting the coordinates of k-th spot.

Let the set of all genes be $G = \{g_1, g_2, \cdots, g_p\}$. We extract the candidate LR pairs for humans from the comprehensively curated database CellChatDB (v.1.1.3) and collect the set of LR pairs denoted as

$$P = \{L_1R_1, L_2R_2, \cdots, L_{\tilde{p}}R_{\tilde{p}} \mid L_i, R_i \in G, 1 \le i \le \tilde{p}\}, \tag{1}$$

where $\tilde{p}$ presents the number of LR pairs ($\tilde{p} = 1,939$ in this work). Next, we filter the overlap genes that play the ligand or receptor role in set P and denote the set of LR genes as,

$$G_{LR} = \{g_1, g_2, \cdots, g_{\hat{p}}\}, \tag{2}$$

where $\hat{p}$ represents the size or cardinality of set G_{LR} ($\hat{p} = 963$ in this work).

Based on this, we can extract their expression profiles $Y \mid_{G_{LR}} \in \mathbb{N}^{n\times \hat{p}}$, where $Y \mid_{G_{LR}}$ is usually called the restriction of matrix Y on the set G_{LR}. By default, FineST exclusively includes ligands and receptors for concentrating on fine-grained LRIs and CCC analysis. Preprocessing scripts:

```
## Selected LR genes. Here gene_list can be ‘LR_genes’, ‘HV_genes’ or ‘LR_HV_genes’
adata = fst.adata_LR(adata, gene_list=‘LR_genes’, species=‘human’)
```

S1.2 Extraction of highly variable gene expression profiles

While our primary objective is to infer cell-cell communication (CCC), and thus our initial analyses centred on LR genes, the FineST framework is not restricted to this gene set. Notably, FineST offers considerable flexibility, enabling the integration of additional gene categories such as highly variable genes (HV genes), marker genes, or any other genes of interest relevant to the biological context under investigation.

To facilitate this flexibility, FineST provides an intuitive interface for gene selection via the function `FineST.adata_LR(adata, gene_list)`, where the `gene_list` argument can be specified as `‘LR_genes’`, `‘HV_genes’`, or `‘LR_HV_genes’`. Here, we demonstrate model input comprising both LR genes and HV genes, implemented through the following preprocessing scripts:

```
## Select LR and/or HV genes; gene_list can be ‘LR_genes’, ‘HV_genes’, or ‘LR_HV_genes’
adata = fst.adata_LR(adata, gene_list=’LR_HV_genes’, species=’human’, n_top_genes=500)
```

S1.3 Spots interpolating

For the ST gene expression data, each sample is a spot with position coordinates. So, with the help of the coordinate representation S, each spot-domain image patch paired with the corresponding sample can be easily awarded position coordination S_k for $1 \le k \le n$. However, as we all know, there is a center-to-center distance (about 100μm) between two adjacent spots in the current 10x Visium platform, which results in a large portion of the tissue being unmeasured for gene expression. In order to address that problem and further predict super-resolved gene expressions outside the original spots (here means “within spots”) as well as in external tissue sections of available histology images, we first construct the virtual spots (also called “between spots”) at the geometric center of any two given spots from two aspects (See **Fig. S2A**, Between spots).

The horizontal direction For each row, we interpolate a new spot between the given spot (x_1, y_1) and the right spot to the given one (x_2, y_2), where the position coordination of the new spot is:

$$(x_H^{1\star 2}, y_H^{1\star 2}) = (\frac{x_1 + x_2}{2}, \frac{y_1 + y_2}{2}). \tag{3}$$

The vertical direction For one row and its up-adjacent row, we select the two closest spots (x_l, y_l) and (x_r, y_r) in the previous row to the given spot (x_o, y_o) in the row and interpolate one new spot in two adjacent rows, where the position coordination of two new spots are:

$$(x_V^{o\star l}, y_V^{o\star l}) = (\frac{x_o + x_l}{2}, \frac{y_o + y_l}{2}), \quad (x_V^{o\star r}, y_V^{o\star r}) = (\frac{x_o + x_r}{2}, \frac{y_o + y_r}{2}). \tag{4}$$

In this manner, the virtual spots are generated to have the same size and a spatial arrangement similar to the real Visium spots. The number of newly interpolated spots is approximately three times that of the original spots, with an interpolation factor of roughly one-fold horizontally and two-fold vertically. Combining both the original and virtual spots yields a total of m spots ($m \cong 4n$), with their coordinates denoted as $S_{\text{all}} \in \mathbb{R}^{m\times 2}$. The preprocessing can be performed using the following scripts:
`position_x = FineST.inter_spot(position, direction = x)`,
`position_y = FineST.inter_spot(position, direction = y)`,
`position_all = FineST.final_pos_list(position_x, position_y, position)`.
Alternatively, the following command can be used:

```
cd ~/FineST
conda activate FineST
python ./demo/Spot_interpolation.py \
    --position_path FineST_tutorial_data/spatial/tissue_positions_list.csv
```

S1.4 Computational constraints for image feature extraction

Computational limitations represent a practical consideration in large-scale image feature extraction. For instance, in typical Visium datasets, the number of `'in_tissue'` spots can approach 5,000. Following sub-spotting, this yields approximately $5,000 \times 4 \times 64$ sub-spots, each with 1,280-dimensional features when utilizing `virchow2` embeddings. We observed that, although FineST can be trained on a single NVIDIA A100-PCIE-40GB GPU, `CUDA out of memory` errors may occur due to the substantial data volume.

To mitigate these memory constraints, we recommend employing the pre-trained `ViT_256` model from HIPT to extract lower-dimensional image embeddings (yielding $5,000 \times 4 \times 16$ sub-spots with 384-dimensional features). This approach alleviates memory usage, albeit with a minor decrease in inference accuracy compared to `virchow2`. This functionality is integrated into the FineST demo, and users can extract image features with the following scripts:

```
cd ~/FineST
conda activate FineST
python ./demo/Image_feature_extraction.py \
    --method Virchow2 \
    --patch_size 112

python ./demo/Image_feature_extraction.py \
    --method HIPT \
    --patch_size 64
```

Comprehensive instructions are provided in the documentation (`https://finest-rtd-tutorial.readthedocs.io/en/latest/HIPT_demo.html`) to facilitate user implementation.

S1.5 Symbolic representation in Moran's R

In particular, for the ligands or receptors that are composed of multiple subunits, we computed the algebraic means as inputs, i.e.,

$$Y_k^L = \frac{\sum_{t_L=1}^{\mathcal{S}_L} Y_k^{t_L}}{\mathcal{S}_L}, \quad Y_s^R = \frac{\sum_{t_R=1}^{\mathcal{S}_R} Y_s^{t_R}}{\mathcal{S}_R}, \tag{5}$$

where $Y_k^{t_L}$ and $Y_s^{t_R}$ present the t_L-th subunit for ligand Y_k^L and the t_R-th subunit for receptor Y_s^R, $\mathcal{S}_L$ and $\mathcal{S}_R$ mean the numbers of ligand subunits and receptor subunits. Besides, in Equation (15), and w_{ks} is the spatial weight matrix calculated by

$$w_{ks} = \frac{M}{\sum_k^M \sum_s^M K_{RBF}(Y_k^L, Y_s^R)} K_{RBF}(Y_k^L, Y_s^R) \tag{6}$$

where $K_{RBF}(Y_k^L, Y_s^R)$ is the RBF kernel function for two spots k and l, which is used to express a measure of similarity between vectors by computing how close they are to each other and can be mathematically represented as follows:

$$K_{RBF}(Y_k^L, Y_s^R) = \exp\left(-\frac{d^2(Y_k^L, Y_s^R)}{2\lambda^2}\right), \tag{7}$$

where $d(\cdot)$ is the Euclidean distance and $d^2(\cdot)$ represents the square of distance $d(\cdot)$, while λ is a parameter that sets the "spread" of the kernel, here we adopt the setting from SpatialDM.

The z-score used to identify significant LRI is defined by

$$z = \frac{R_k^{Local} - 0}{\sqrt{\mathrm{Var}(R_k^{Local})}}, \tag{8}$$

where the $\mathrm{Var}(R_k^{Local})$ in Equation (8) is the variance for R_k^{Local} represented by

$$\mathrm{Var}\left(R_k^{Local}\right) = 2\frac{(M-1)^2}{M^2}\sigma_k^2\sigma_l^2\sum_{l=1}^{n} w_{kl}^2 + 2\frac{(M-1)^2}{M^2}\sigma_k^2\sigma_l^2 w_{kk}^2, \tag{9}$$

The detailed calculation and derivation process can be found in the Supplementary material in SpatialDM [2].

S1.6 Visualization of sender and receiver cell types for LRIs

In FineST, the spatial communication score (SCS) parameter can be flexibly chosen from several options, including the default `'P_value'`, as well as `'R_local'`, `'sender'`, or `'receiver'`. Notably, the `'R_local'` option employs the bivariate local Moran's R statistic, as detailed in **Equation** (16) of the FineST manuscript, to provide a more refined evaluation of spatial interactions. When this setting is applied, sender and receiver cell types are distinctly visualized, resulting in more intuitive and informative representations of ligand–receptor interactions. Preprocessing scripts:

```
import FineST as fst
import FineST.plottings as fstplt
fstplt.plot_pairs_dot_class(adata, 'DLL4_NOTCH4', SCS='R_local').
```

S2 Supplementary Tables

Table S1. The datasets analyzed in FineST.

Tissue	Sample	Protocol	Data source	Data dim	HE image	Spot diam	Reference
**CRC**	Sample P2 CRC	10x Visium HD	`https://www.10xgenomics.com/products/visium-hd-spatial-gene-expression/dataset-human-crc`	16μm: 137,051 bins; 18,085 genes 8μm: 545,913 bins; 18,085 genes	Visium_HD_Human_Colon_Cancer_tissue_image.btf (10.3GB)	≈ 58 pixels	[3]
**CRC**	Chromium Single Cell Flex, aggregated	10x Single Cell Gene Expression Flex	`https://www.10xgenomics.com/products/visium-hd-spatial-gene-expression/dataset-human-crc`	279,691 cells; 18,167 genes	/	/	[3]
**BRCA**	GSM7782699	10x Visium v1	`https://www.ncbi.nlm.nih.gov/geo/query/acc.cgi?acc=GSE243280`	4,992 spots; 18,085 genes	GSM7782699_tissue_image.tif (1.2GB)	≈ 128 pixels	[4]
**BRCA**	GSM7780153	10x Xenium	`https://www.ncbi.nlm.nih.gov/geo/query/acc.cgi?acc=GSE243280`	167,780 cells; 313 genes	morphology.ome.tif (4.8GB)	/	[4]
**BRCA**	GSM7782698	10x Single Cell Gene Expression Flex	`https://www.ncbi.nlm.nih.gov/geo/query/acc.cgi?acc=GSE243280`	27,460 cells; 18,082 genes	/	/	[4]
**HCC**	P1T_Spatial	10x Visium v1	`https://data.mendeley.com/datasets/skrx2fz79n/1`	3,348 spots; 36,601 genes	T11-V10F06-115-A1.jpg (52.5MB)	≈ 130 pixels	[1]
**HCC**	P7T_Spatial	10x Visium v1	`https://data.mendeley.com/datasets/skrx2fz79n/1`	4,106 spots; 36,601 genes	liuyao-T9-V10M02-092-B1.jpg (57.6MB)	≈ 130 pixels	[1]
**NPC**	GSM6030901 NPC_patient_1	10x Visium v1	`https://www.ncbi.nlm.nih.gov/geo/query/acc.cgi?acc=GSE200310`	1,331 spots; 36,601 genes	20210809-C-AH4199551.tif (862.6MB)	≈ 139 pixels	[5]
**NPC**	Integrated scRNA-seq data	/	/	5,508 cells; 33,628 genes	/	/	[5]
**HER2+ BRCA**	Section G1	Spatial Transcriptomics	`https://github.com/almaan/her2st`	441 spots; 14,992 genes	HE_BT23903_C1a.jpg (25.3MB)	≈ 400 pixels	[6]

Note: **CRC**: Colorectal cancer; **HCC**: Hepatocellular carcinoma; **NPC**: Nasopharyngeal carcinoma; **BRCA**: Breast cancer; **HER2+ BRCA**: HER2-positive breast cancer; **dim**: dimension; **diam**: diameter.

Table S2. Sparsity in representative VisiumHD datasets.

Dataset	ST	Scale	# squares	# genes	Ratio of zeros
CRC [3]	Visium v1	89.439 pixel	2,702 spots	32,285	81.622%
CRC [3]	Visium HD	16μm	137,051	18,085	92.951%
		8μm	545,913		97.826%
		2μm	8,731,400		99.841%
Small Intestine (Date Published: 2024-03-26)		16μm	91,033	19,059	94.929%
		8μm	351,817		98.400%
		2μm	5,479,660		99.877%
Mouse Brain (Date Published: 2024-03-30)		16μm	98,917	19,059	96.379%
		8μm	393,543		98.942%
		2μm	6,296,688		99.927%

https://www.10xgenomics.com/datasets/visium-hd-cytassist-gene-expression-libraries-of- [mouse-intestine] or [mouse-brain-he]

Table S3. The supporting of FineST's identified two key LR pairs in previous studies.

Subtype	Ligand 1	Ligand 2	Receptor 1	Receptor 2	Signal	Function	Description
	**NRG1**		**ERBB3**				
DCIS 1	Mainly stroma, low expression in tumor cells		Low-medium		Paracrine	Promote mild growth, limited activity	HER3 is highly expressed in luminal breast cancer subtypes. Its activation by NRG1 promotes activation of AKT and ERK1/2, contributing to tumour progression and therapy resistance. [7]
DCIS 2	Stromal + some tumor cells		Medium-high		Paracrine or autocrine	Enhanced growth, survival, and progression	NRG1 and ERBB3 overexpression exacerbate tumor growth and progression. NRG1–ERBB3 interaction interface is an interesting target for anti-tumor drug development to block tumor progression. [8]
Invasive tumor	Stromal + high expression in tumor cells		High		Paracrine or autocrine	Invasion, metastasis, and drug resistance	ERBB3 expression correlates with increased intravasation and metastasis, with metastatic samples exhibiting higher ERBB3 than primary tumors. [9]
	**INHBA**	**INHBB**	**ACVR1C**	**ACVR2A**			
DCIS 1	Low-medium (mostly stroma)	Low	Low	Low-medium	Paracrine	Cell proliferation, mild EMT	INHBA is associated with aggressiveness of the basal subtype of HER2+ tumors, patients with HER2+ breast cancer and high levels of INHBA expression had worse outcomes than patients with low levels of INHBA expression. [10]
DCIS 2	Medium-high (Upregulated in some tumor cells)	Medium	Medium-high	Medium-high	Paracrine or autocrine	Promote EMT and enhance invasive potential	In breast cancer, ACVR1C expression was inversely correlated with the tumor grade and clinical stage. [11]
Invasive tumor	High (Highly expressed in tumor cells)	High	High	High	Paracrine and autocrine	Promote invasion, metastasis, and drug resistance	INHBA and INHBB are strongly expressed in breast cancer and high expression of INHBA correlates with poorer survival. [12]

Table S4. The comparison of FineST and iStar that run with the same setup.

Datasets	Sample	Ground truth	# genes			PCC of genes			w
			LR	HV	ALL	iStar	FineST inferred	FineST imputed	
**CRC**		Visium HD	862			0.092		0.507	
**NPC**		Visium spot	596			0.293		0.990	0.50
**HCC**	P1_T_Spatial	Visium spot	911	500	1,073	0.201	0.221	0.630	0.02
	P7_T_Spatial	Visium spot	911	500	1,124	0.209	0.247	0.567	
**BRCA**	Visium	Visium spot	864	100	957	0.404	0.440	0.589	0.02
	Xenium adjacent	Xenium psudo-spot			65	0.543	0.601	0.616	
**HER2+**	iStar demo	ST spot	647			0.330	0.518		0.00

Table S5. Top 20 features of SPPI+ macrophage and CAF from literature [1].

**SPPI_macrophage**	**CAF**
CSTB	LUM
SPP1	VCAN
FTL	TIMP1
FABP5	TMSB10
CTSD	COL1A2
RNASE1	COL6A3
GPNMB	CTHRC1
LGALS1	CCL4
TM4SF19	ZFP36L2
NUPR1	COL1A1
CTSL	EFEMP1
LGALS3	SPON2
CCL7	THY1
VIM	F2R
SLAMF9	LAMA2
FABP4	S100A10
BNIP3	ITGBL1
MIF	ALB
ATP6V1F	CXCR4
CD68	STK17B

Table S6. The pre-trained Vision Transformer (ViT) used in FineST.

**ViT**	**Released**	**URL**	**# WSIs**	**Architecture**	**Input size**	**Tile size**	**Embed dim**	**Reference**
`Virchow2`	Aug, 2024	`https://huggingface.co/owkin/phikon-v2`	3.1M	ViT-H/14	224 pixels	14 pixels	1,280	[13]
`vit256-16`	Jun, 2022	`https://github.com/mahmoodlab/HIPT`	10,678	ViT-S/16	256 pixels	16 pixels	384	[14]

Table S7. Significant LR pairs detected in Visium HCC ROI.

	**P1_T_Spatial**		**P7_T_Spatial**	
**# genes**	1073		1124	
**# spots**	393		744	
**# spots_selected**	198		648	
**# LR pairs**	573	555 (≥ 3)	583	583 (≥ 3)

Note: The notation ≥ 3 denotes that the LR interaction is present at more than three spots.

Table S8. The software compared with FineST.

Method	Published	URL	Reference
**iStar**	May 9, 2023	https://github.com/daviddaiweizhang/istar	[15]
**TESLA**	Jan 2, 2024	https://github.com/jianhuupenn/TESLA	[16]

S3 Supplementary Figures

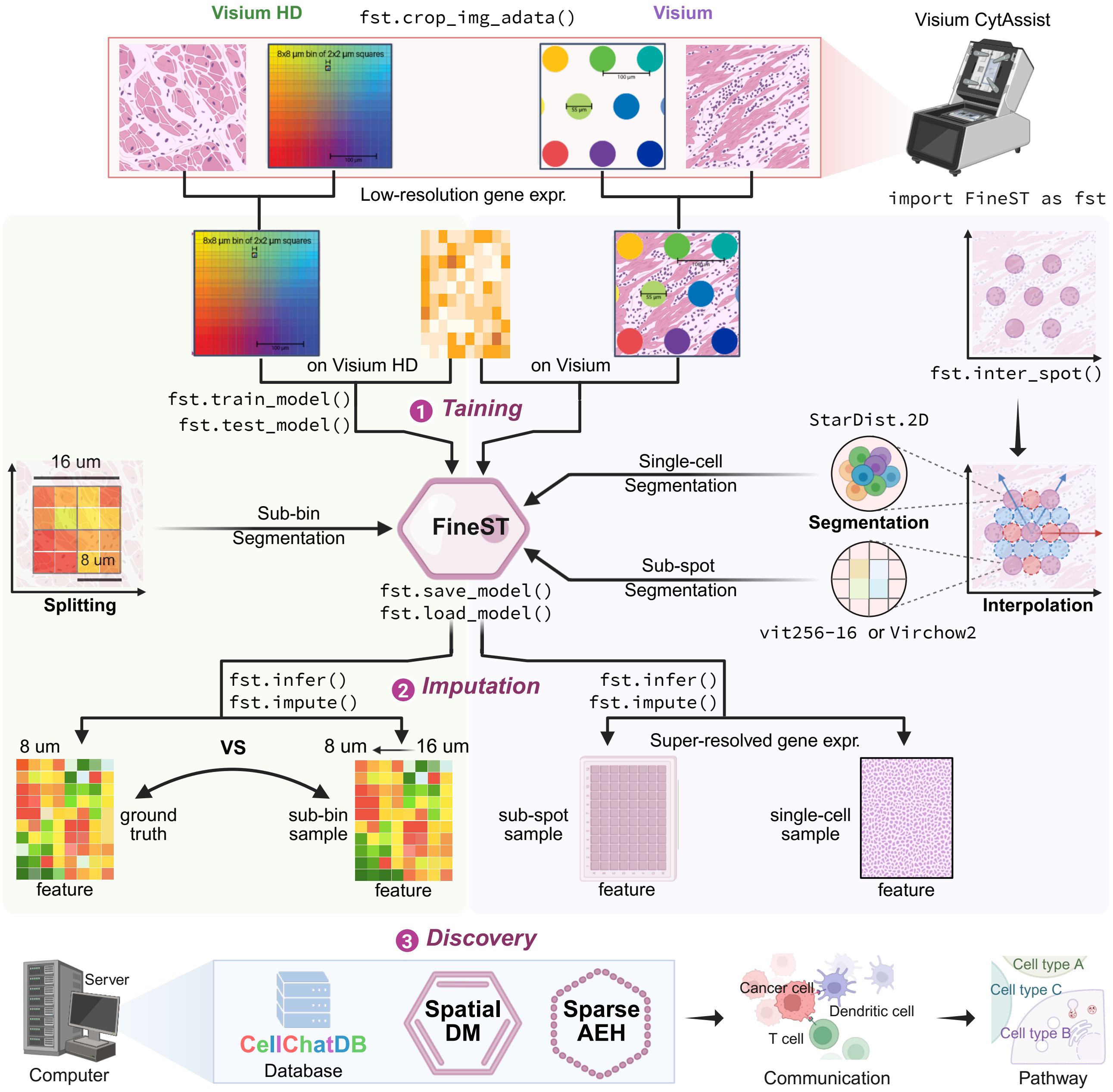

Figure S1. The overview of FineST (<u>Fine</u>-grained <u>S</u>patial <u>T</u>ranscriptomics). FineST adopts a bimodal contrastive learning model to utilize spatial gene expression and corresponding histological image, enabling super-resolved gene expression among captured and unmeasured areas across the whole slide imaging (WSI). The *Training-Imputation-Discovery* module induced by FineST, together with SpatialDM and SparseAEH, could greatly enhance cell type annotation in spatial cellular resolution, ligand-receptor interaction discovery and cell-cell communication detection in spatial sub-spot or single-nuclei resolution (Methods). This figure is created in BioRender. Huang, Y. (2026) `https://BioRender.com/5ak7zco`.

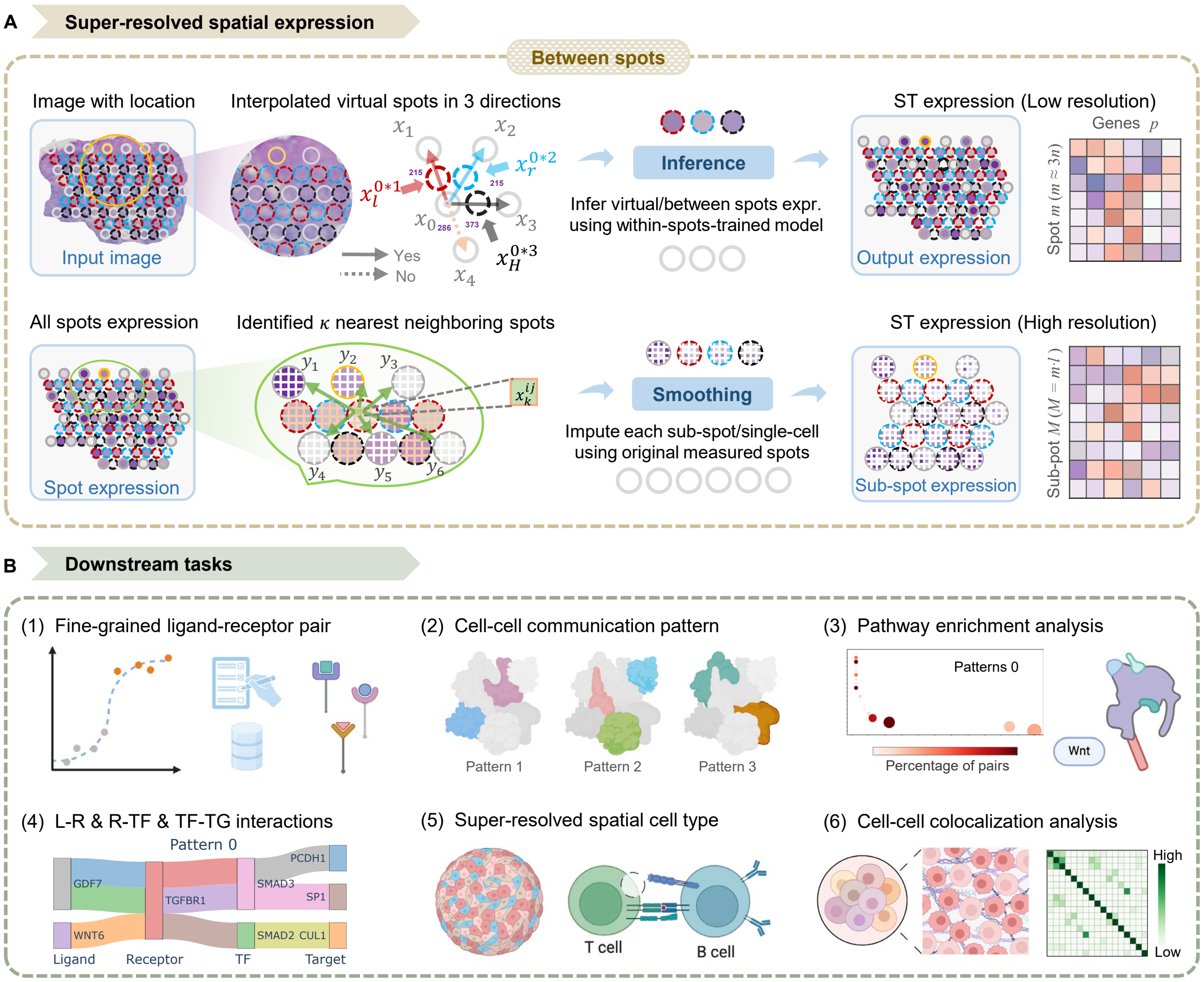

Figure S2. The imputation and downstream tasks of FineST. (A) Inference and imputation of FineST. Once the model training finishes (**Fig. 1B**), the inference can be performed for patch images between spots. Then the imputation of sub-spot or single-cell can also be achieved by averaging the inference from the image and smoothing from κ neighboring measured spots to get a higher-resolution expression. **(B)** The six primary utility functions in the FineST toolbox for downstream analysis include (1) Fine-grained ligand-receptor pair discovery, (2) Cell-cell communication pattern clustering, (3) Pathway enrichment analysis, (4) Ligand-receptor (L-R), receptor-transcription factor (R-TF), and transcription factor-target genes (TF-TG) interactions, (5) Super-resolved spatial cell type annotation and (6) Cell-cell co-localization analysis. Part of panel **(B)** is created in BioRender. Huang, Y. (2026) `https://BioRender.com/1mns9p1`.

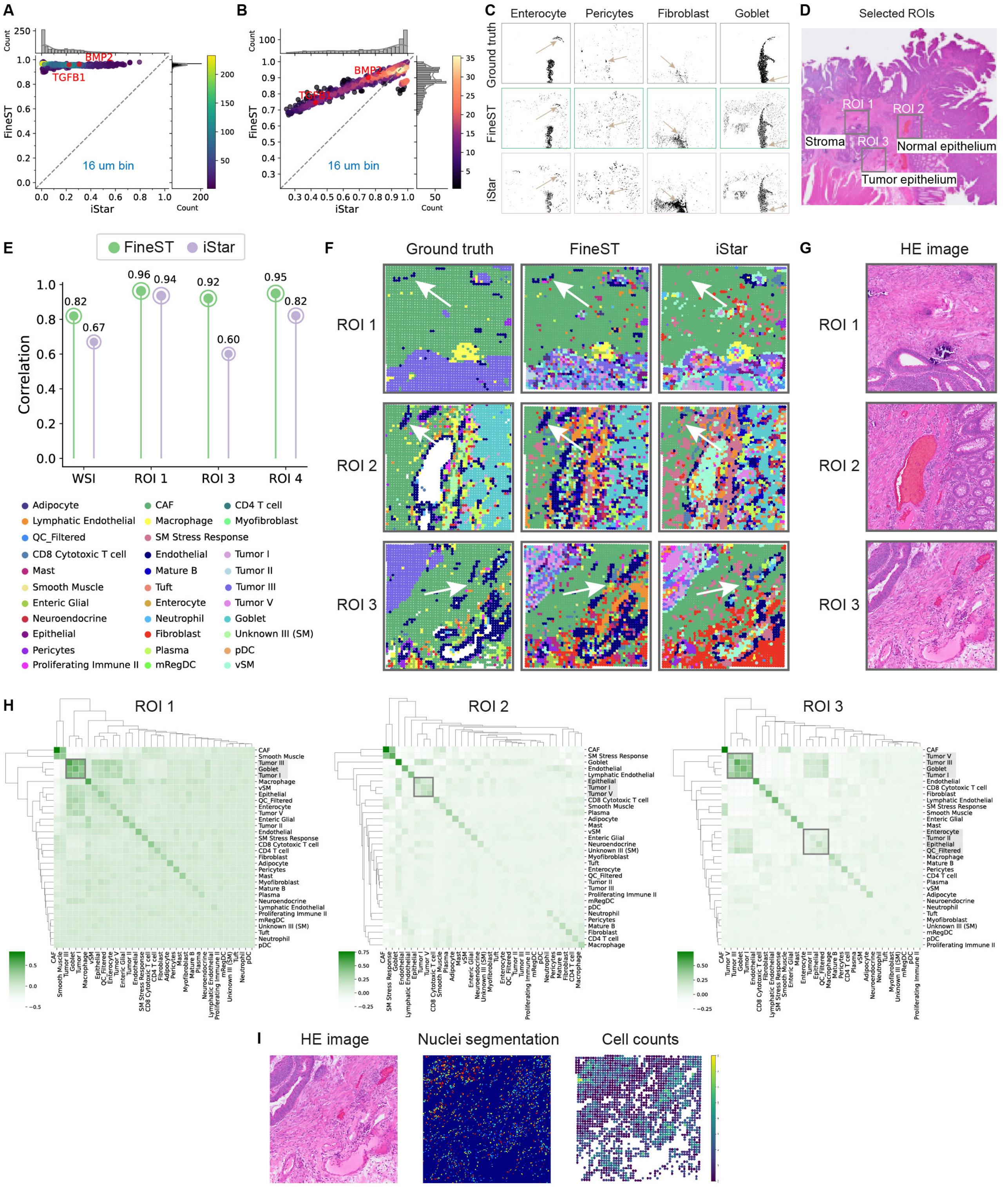

Figure S3. FineST achieved accurate prediction and pinpointed cell types on the VisiumHD CRC dataset with 16μm resolution. (A-B) Scatter plot of Pearson correlation **(A)** and structural similarity index **(B)** (FineST vs iStar) between the original gene expressions and the predictions made by FineST for 862 LR genes across the filtered 136,954 squares out of 137,051 squares. **(C)** Four cell types associated with Rec. 1, Rec. 2 and Rec. 3 in **Fig. 2G,H**. The results underscore the superior performance of FineST in deconvoluting results compared to iStar. **(D)** HE image with three annotated ROIs. **(E)** Numeric comparison based on the Pearson coefficients of cell type proportion between (FineST vs iStar) and ground truth. **(F)** Spatial cell type deconvolution results within each ROI of FineST and iStar, compared with the ground truth. **(G)** Zoom-in view of three selected ROIs from HE image. **(H)** Cell type co-localization for each ROI depicted in **D**. The cluster maps are color-coded based on the bivariate statistic Moran's R for different pairs of cell types. **(I)** Nuclei segmentation results within ROI 3. For panels **(A)**, **(B)** and **(E)**, source data are provided as a Source Data file.

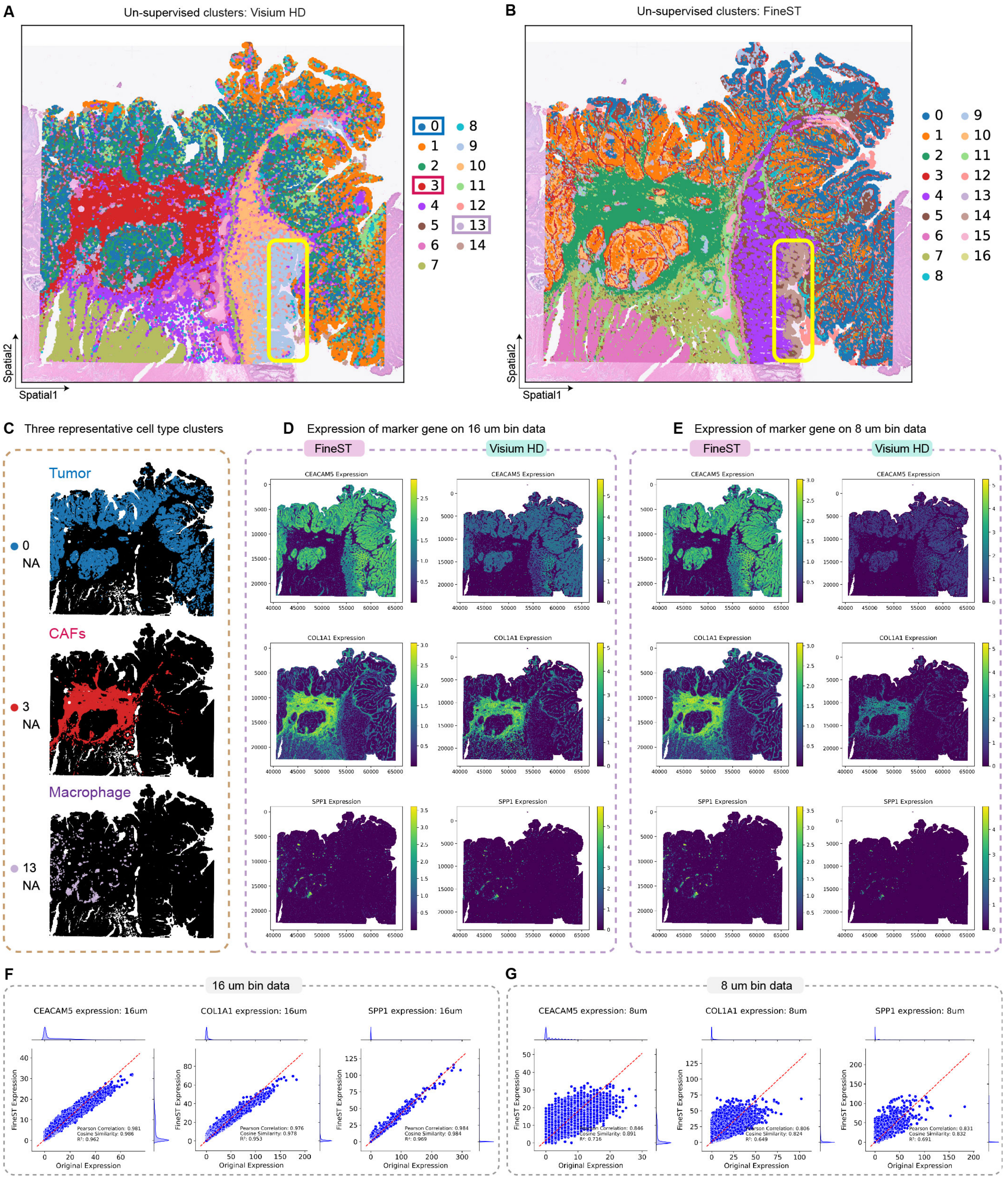

Figure S4. FineST facilitated more refined cell clusters on the VisiumHD CRC dataset with 16μm resolution. (A) Unsupervised clustering of cell types was performed using the original VisiumHD data. **(B)** Unsupervised clustering of cell types was performed using the imputed data from FineST. FineST facilitates a more detailed spatial co-localization of cell types and tissues, especially for the yellow rectangle region, compared to the ground truth presented in **Fig. 2H**. **(C)** Three representative cell types from **A**, namely cluster 0, cluster 3, and cluster 13. **(D)** The marker gene expressions correspond to three cell types in **C** on 16μm resolution. **(E)** The marker gene expressions correspond to three cell types in **C** on 8μm resolution. The gene expression predicted by FineST (Left) exhibits a stronger correlation compared to the original VisiumHD gene expression (Right). **(F-G)** The numeric comparison of FineST's imputed gene expression and the original gene expression.

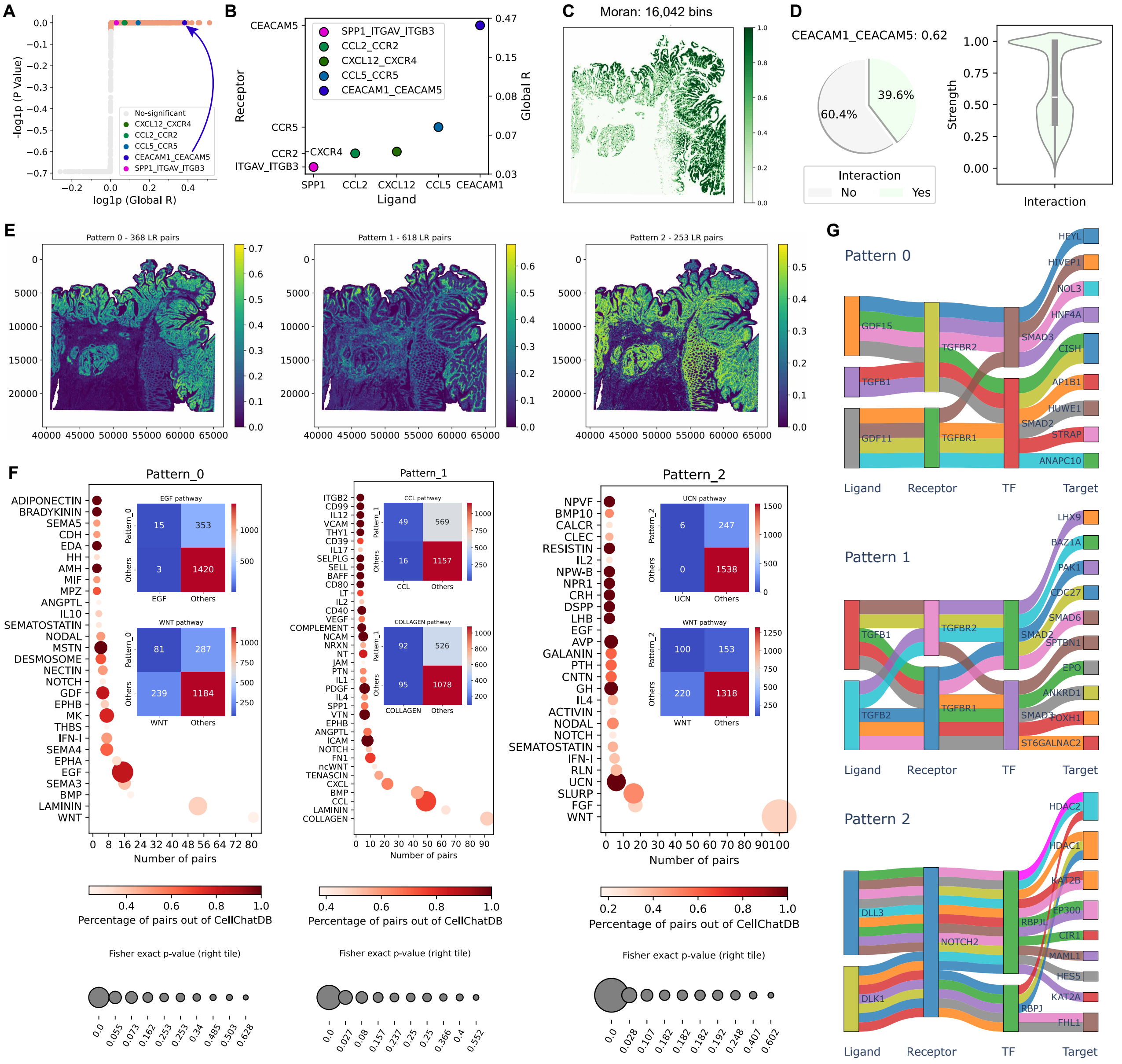

Figure S5. FineST discovered intricate ligand-receptor pairs and cell-cell communication patterns on CRC dataset with 16μm resolution. **(A)** Scatter plot of global Moran's R underlying z-score p-value, where the x-axis is log(Global Moran's R+1), y-axis is $-\log(p \text{ value}+1)$. Here 1,253 spatially co-expressed LR pairs are identified as significant (FDR $<$ 0.05) and highlighted in orange, otherwise gray. Source data are provided as a Source Data file. **(B)** The five representative LR pairs, as shown in **A**, where the x-axis is a ligand, the left y-axis is a receptor and the right y-axis is the global Moran's R value R^{Global} corresponding to each LR pair (i.e., each dot). **(C)** The interaction of *CEACAM1-CEACAM5* pair was observed in 16,042 bins, which maps the tumor region as shown in **Fig. 2H**. **(C)** Considering the 1,239 LR pairs that are active in more than 22 bins (by taking the percentile as threshold), it shows the *CEACAM1-CEACAM5* interacts among 39.6% bins. The average interaction strength of *CEACAM1-CEACAM5* pair is 0.62, quantify using local Moren's R value R_k^{Local}. **(E)** Three patterns were enriched using the selected 1,239 LR pairs, where Pattern 0 and Pattern 2 mapped the tumor region (Tumor I and Tumor II, respectively), while Pattern 1 maps the CD4 T cell region, by checking the ground truth. **(F)** Dot plots of enriched pathways of three patterns, in which Pattern 0 and Pattern 2 are significantly enriched by the WNT pathway. The y-axis represents the name of the enriched pathway, and the x-axis represents the number of significant LR pairs in that pathway for each pattern. The dot color shows the percentage of significant pairs for that pathway from CellChatDB, while the dot size shows the significance of enrichment from the two-sided Fisher's exact test. The confusion matrix shows the 2-by-2 contingency table describing the pathway that belongs to its corresponding pattern. **(G)** Certain LR pairs with their downstream transcript factors from DcjComm [17] and corresponding target genes from RegNetwork [18] correspond to each pattern.

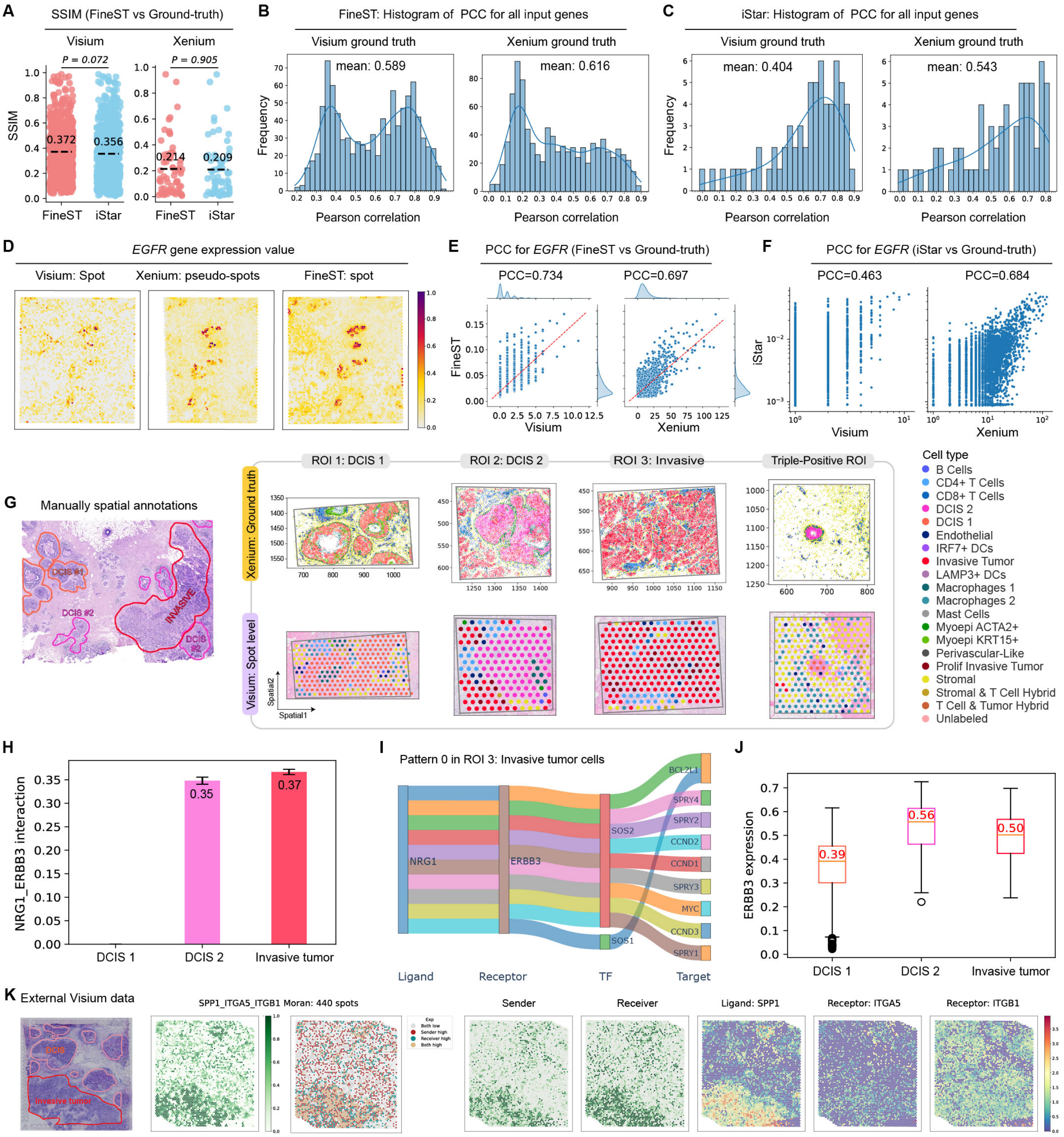

Figure S6. FineST outperforms iStar in gene expression prediction and identifies biologically relevant ligand-receptor pairs at the single-cell level. **(A)** SSIM index of FineST and iStar with Viisum or Xenium as ground truth. Source data are provided as a Source Data file. **(B-C)** Histogram of Pearson correlation coefficients obtained from FineST **(B)** and iStar **(C)** for all input genes (957 genes across 4,992 Visium spots, 65 overlapping genes in 3,958 Xenium pseudo-spots). **(D-F)** Visualization and comparison of iStar Pearson correlation for the selected marker gene *EGFR*, each dot represents a Visium spot (n =4,992) or overlapping Xenium pseudo-spots (n =3,958). **(G)** Selected cell subtypes in Xenium with manual spatial annotation on HE images (from literature [4]), followed by cell composition within each ROI at single-cell (Xenium) and spot (Visium) levels, as reported in the literature [4]. **(H)** Barplot of *NRG1-ERBB3* interaction across DCIS 1, DCIS 2 and Invasive tumor cells. Note that there is an absence of significant interaction in DCIS 1. **(I)** Sankey plot illustrating the *NRG1-ERBB3* interaction and its downstream CCC pathway (L-R-TF-TG) in Pattern 0 of Invasive tumor cells. **(J)** *ERBB3* expression levels within three ROIs (3,302 cells in DCIS 1; 1,208 cells in DCIS 2; 3,943 cells in Invasive tumor cells), with the median indicated by a red line. **(K)** Independent dataset validation result of *SPP1-ITGA5-ITGB1* interaction.

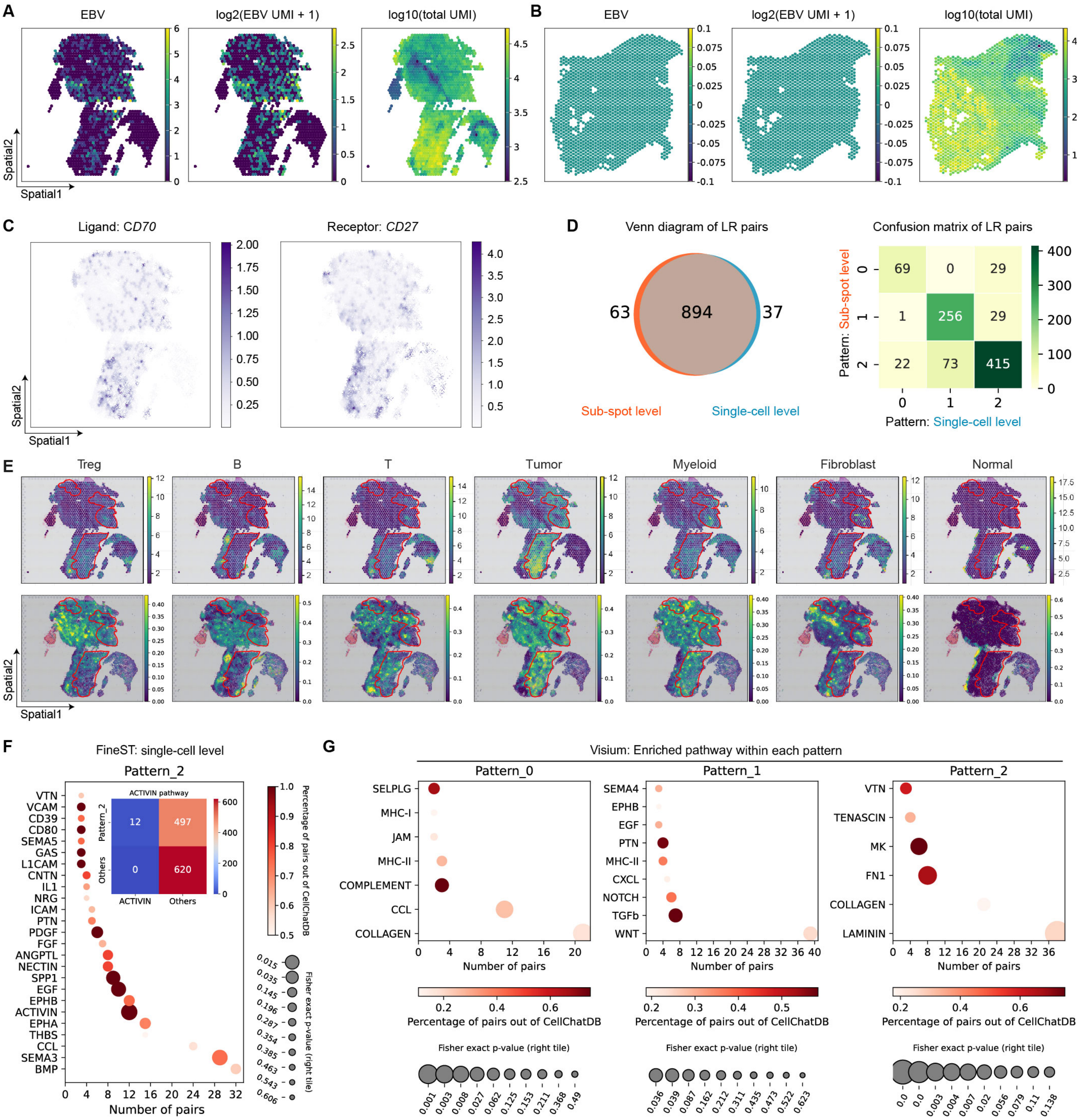

Figure S7. FineST detected fine-grained ligand-receptor interaction on the Visium NPC dataset. **(A)** The viral transcripts (in UMI) of Epstein-Barr virus are widely detected in NPC patient1 sample, consistent with the original literature [5]. **(B)** The negative control: Prostate sample P2_H2_5 from literature [19]. **(C)** Expression of ligand *CD70* and receptor *CD27* in spatial local single-cell from **Fig. 3K**. **(D)** Left: Overlap of LR pairs obtained from two types of super-resolution local CCC detection approaches. Right: Pattern consistency analysis between sub-spot level and single-cell level. Source data are provided as a Source Data file. **(E)** Top: The spatial co-localization of seven cell types in NPC tissues, estimated by cell2location, provided by the original study. Bottom: The cell co-localization of seven cell types deconvoluted using TransImp from FineST's imputed single-cell resolution gene expression. **(F)** Dot plot showing enriched pathways in Pattern2 at single-cell resolution (**Fig. 4L**, Bottom), with significant enrichment of the ACTIVIN pathway. **(G)** Dot plot of enriched pathways detected by 10x Visium at spot resolution.

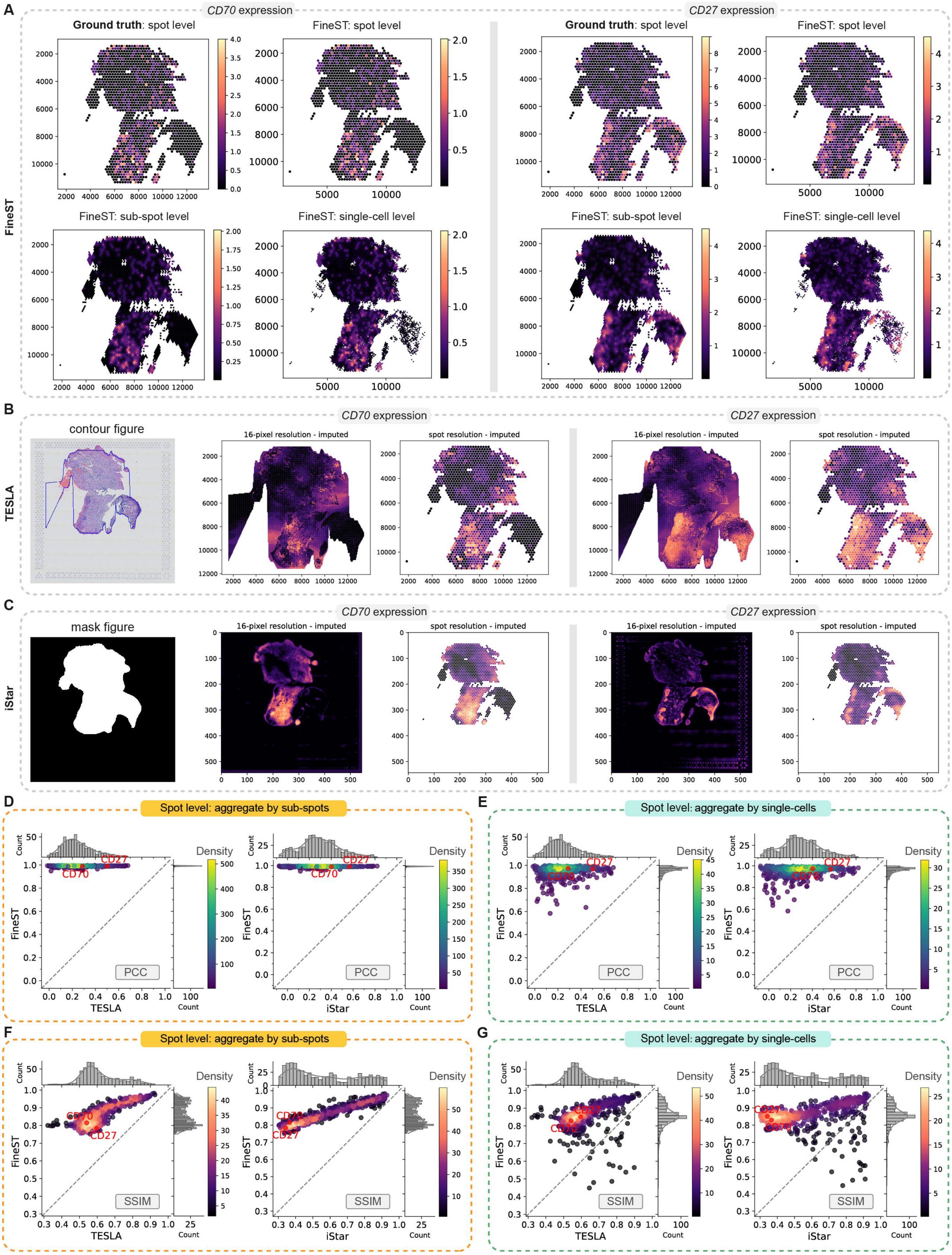

Figure S8. Super-resolved ST expression profile evaluation on the Visium NPC dataset. **(A)** Gene expression of *CD70* and *CD27* on NPC cells at original spot level (with $n = 1,331$ spots) and super-resolution level (with $M = 322,496$ sub-spots aggregated in 5,039 spots and $\hat{M} = 40,068$ single cells). **(B-C)** TESLA's detected contour (**B**) and iStar's mask contour (**C**) of tissue in HE image and their imputed gene expression at high-resolution and spot-resolution. **(D-G)** Comparisons of Pearson correlation (**D, E**) and structural similarity index (**F, G**) for FineST, TESLA, and iStar methods for sub-spot segmentation and single-cell segmentation enhancement of gene expression, where each dot represents one of the 596 LR genes. For panels (**D**), (**E**), (**F**) and (**G**), source data are provided as a Source Data file.

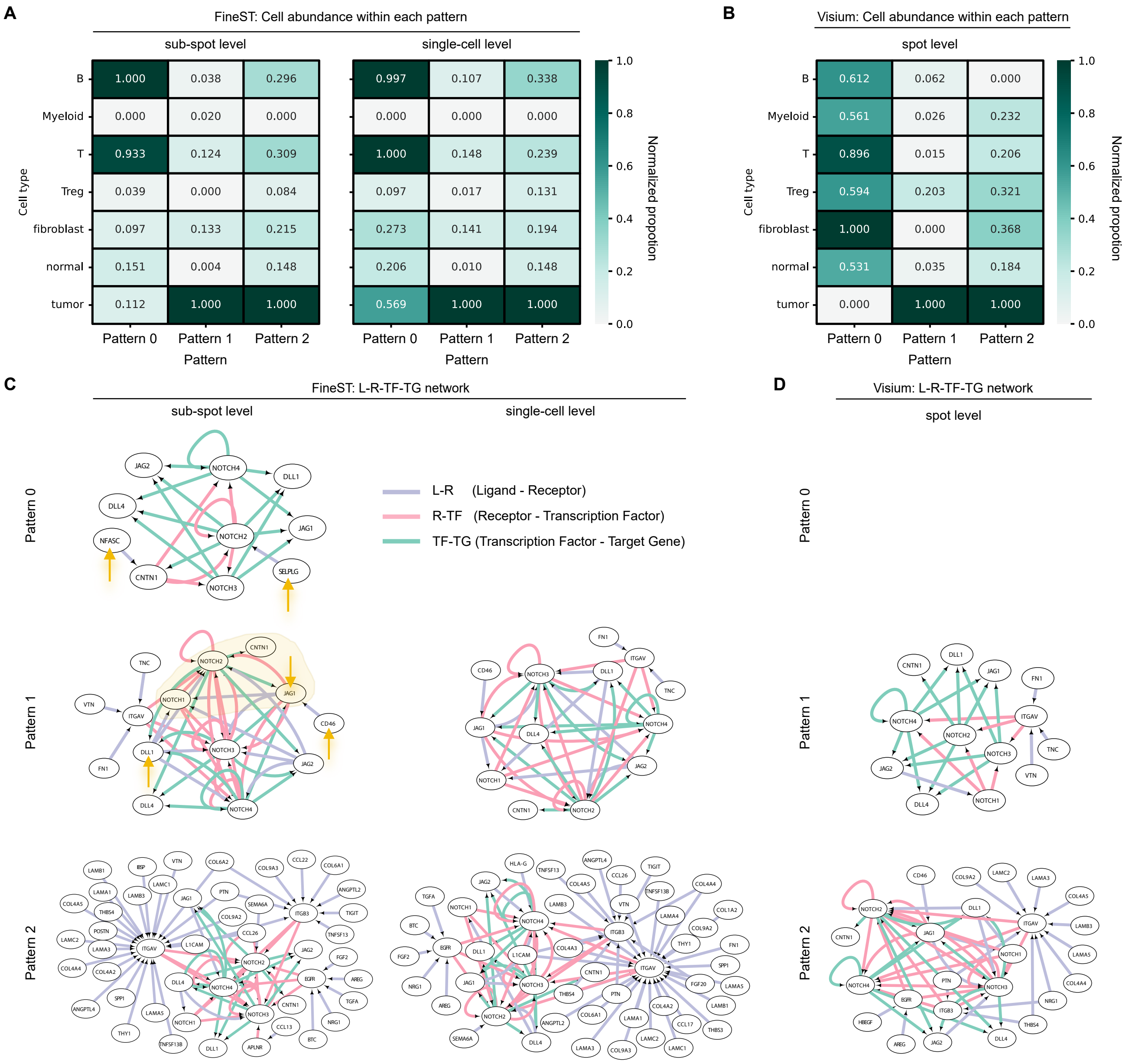

Figure S9. FineST-identified LR pairs are biologically meaningful and mechanistically distinct, highlighting the value of high-resolution CCC analysis. (A-B) Heatmaps of cell abundance associated with each spatial pattern. FineST reveals that Pattern 0 is enriched for B cells and T cells, whereas Visium does not show significant T cell enrichment. **(C-D)** L-R-TF-TG (Ligand–Receptor–Transcription Factor–Target Gene) network module analysis. FineST identifies a unique regulatory network module in Pattern 0 (immune cell-enriched), driven by two ligands (*NFASC*, *SELPLG*), which are not detected at the lower Visium resolution. Source data are provided as a Source Data file.

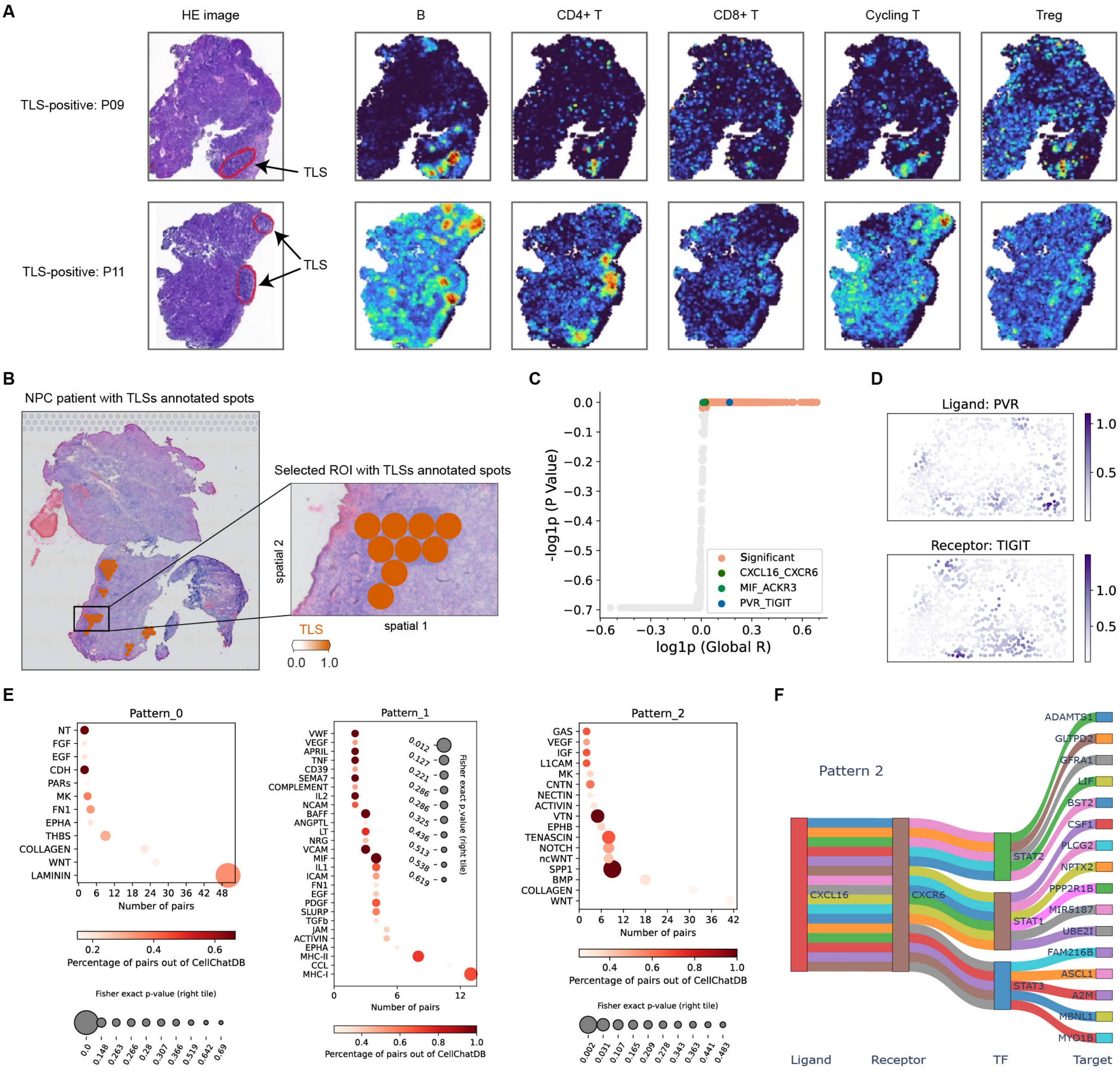

Figure S10. FineST achieved region-specific fine-grained ligand-receptor interaction within selected ROIs. (A) Pathologists annotated tertiary lymphoid structures (TLSs) regions on HE slides (red circles). You et al. [20] showed that B cells are localized within TLSs, and their colocalization analysis further revealed that CD4+ T cells, CD8+ T cells, cycling T cells, and Treg cells are generally located closer B cells in primary NPC [20] (Note: Data are adapted from You et al. and used here to support our findings). **(B)** Pathologists have identified 36 spots (out of a total of 1,331 spots) that are co-localized with tertiary lymphoid structures (TLSs), which could enhance tumor antigen presentation and T cell activation. For the zoom-in view, the presence of TLSs from selected ROI was further validated by the co-location of T cells and B cells at single-cell level from FineST, as evidenced by **Fig. 4B"**. **(C)** Scatterplot of global Moran's R underlying z-score p-value from the selected region of interest in **Fig. 4B"**. Source data are provided as a Source Data file. **(D)** Expression of ligand *PVR* and receptor *TIGIT* in spatial local single-cell from **Fig. 4G**. **(E)** Dot plots of enriched pathways in three patterns from **Fig. 4H**, where Pattern 2 enriched WNT pathway. **(F)** Sankey plot of CCC pathways of significant L-R-TF-TG in Pattern 2, here *CXCL16*-*CXCR6* pair is demonstrated.

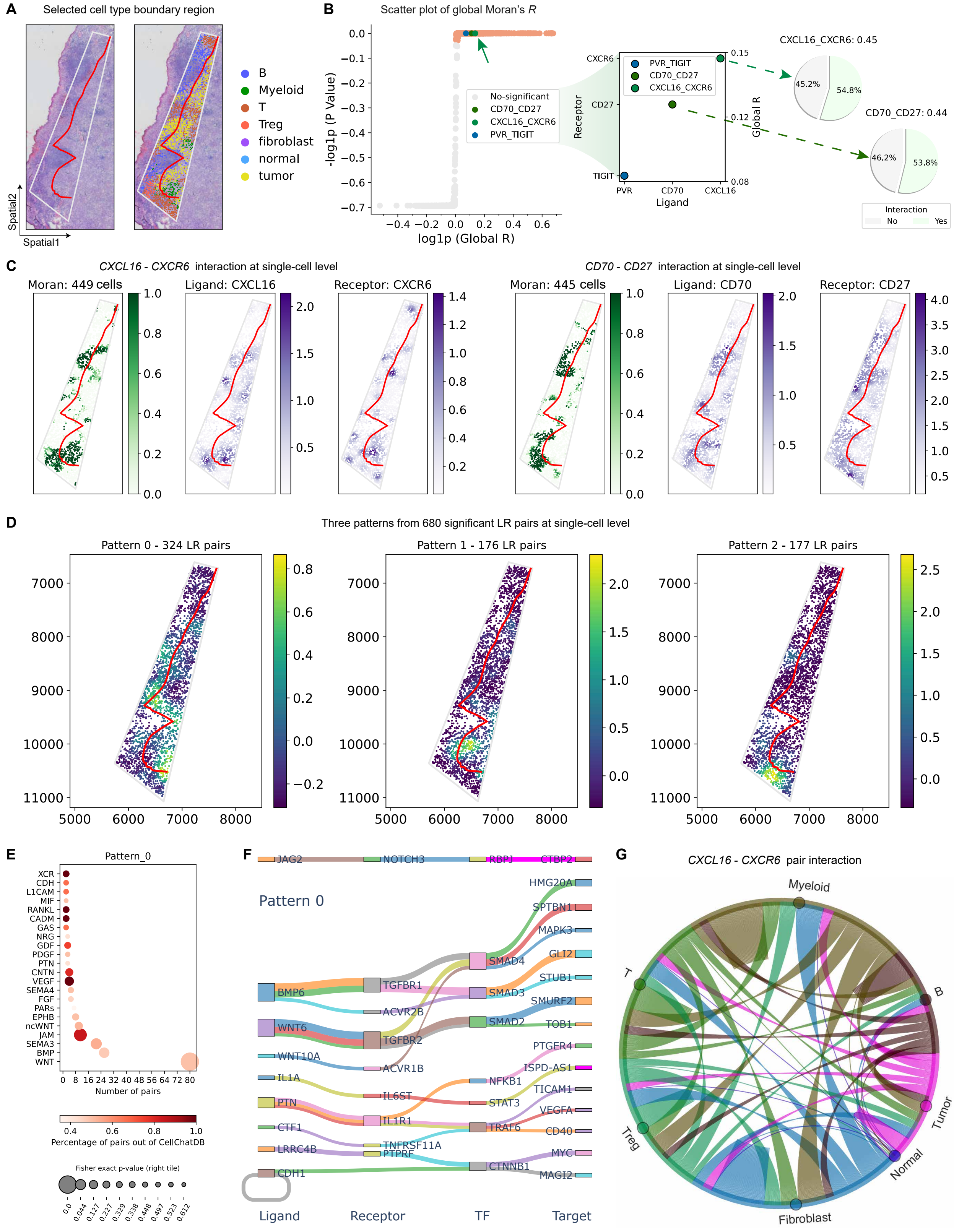

Figure S11. FineST discovered fine-grained LR interaction at the boundary of Tregs and Tumor cells. (A) The ROI contains the boundary of Treg and NPC tumor cells at single-cell resolution, where the red line is from **Fig. 4I**. **(B)** Scatter plot of global Moran's R and the p-value of z-score approach, where 677 significant pairs are selected. Source data are provided as a Source Data file. **(C)** The selected local single-cell of *CXCL16-CXCR6* pair and *CD70-CD27* pair with corresponding gene expression in the spatial domain. **(D)** Clustering of 677 selected LR pairs with interactions in more than 2 cells into three spatial patterns by SpatialDE. Each plot is colored by the posterior mean of local statistics in each spatial pattern. **(E)** Dot plots of enriched pathways in Pattern 0, which maps the boundary of Tregs and Tumors, as well as TLS shown in **Fig. 4E**. **(F)** Sankey plot of CCC pathways of significant L-R-TF-TG in Pattern 0. **(G)** Chord diagram summarizing cell types interacting for *CXCL16-CXCR6*.

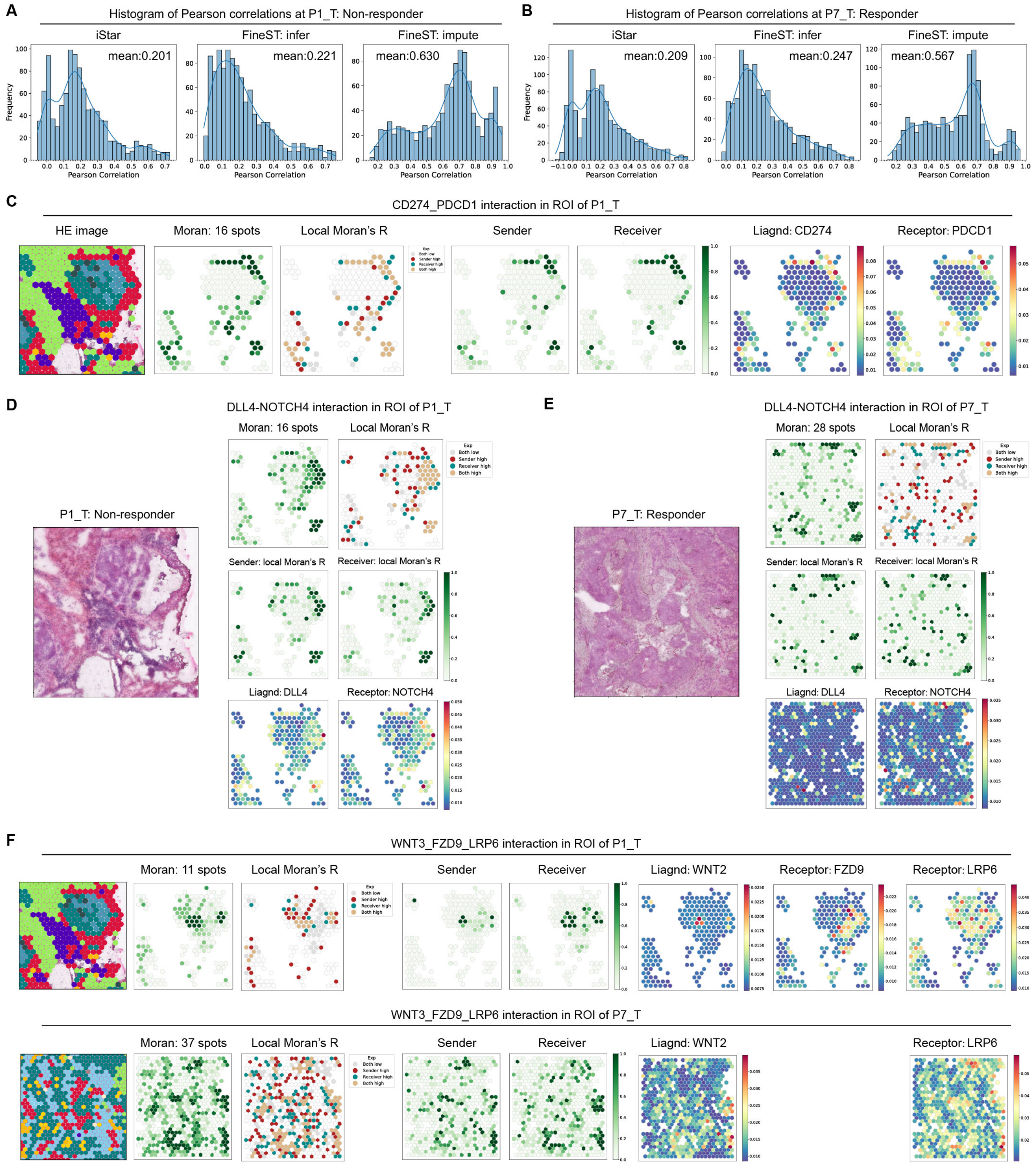

Figure S12. FineST outperforms iStar in gene expression prediction and uncovers putative cellular crosstalk within tumor-immune barrier in HCC. **(A-B)** Histogram of Pearson correlation coefficients of FineST and iStar on non-responder **(A)** and responder **(B)** for all input genes (1,073 genes across 3,348 Visium spots for P1_T, 1,124 overlapping genes in 4,106 Visium spots for P7_T). **(C)** The immunosuppressive interaction between *CD274* and *PDCD1* is present only in ROI1 of non-responders and is not observed in responders. **(D-E)** The cropped HE image of two ROIs selected from P1_T and P7_T, and spatial interaction and gene expression pattern of the *DLL4–NOTCH4* pair in ROI of P1_T (non-responder) and P7_T (responder). **(F)** Spatial plot illustrating the *WNT3-FZD9-LRP6* interaction and its gene expression in non-responder and responder patients.

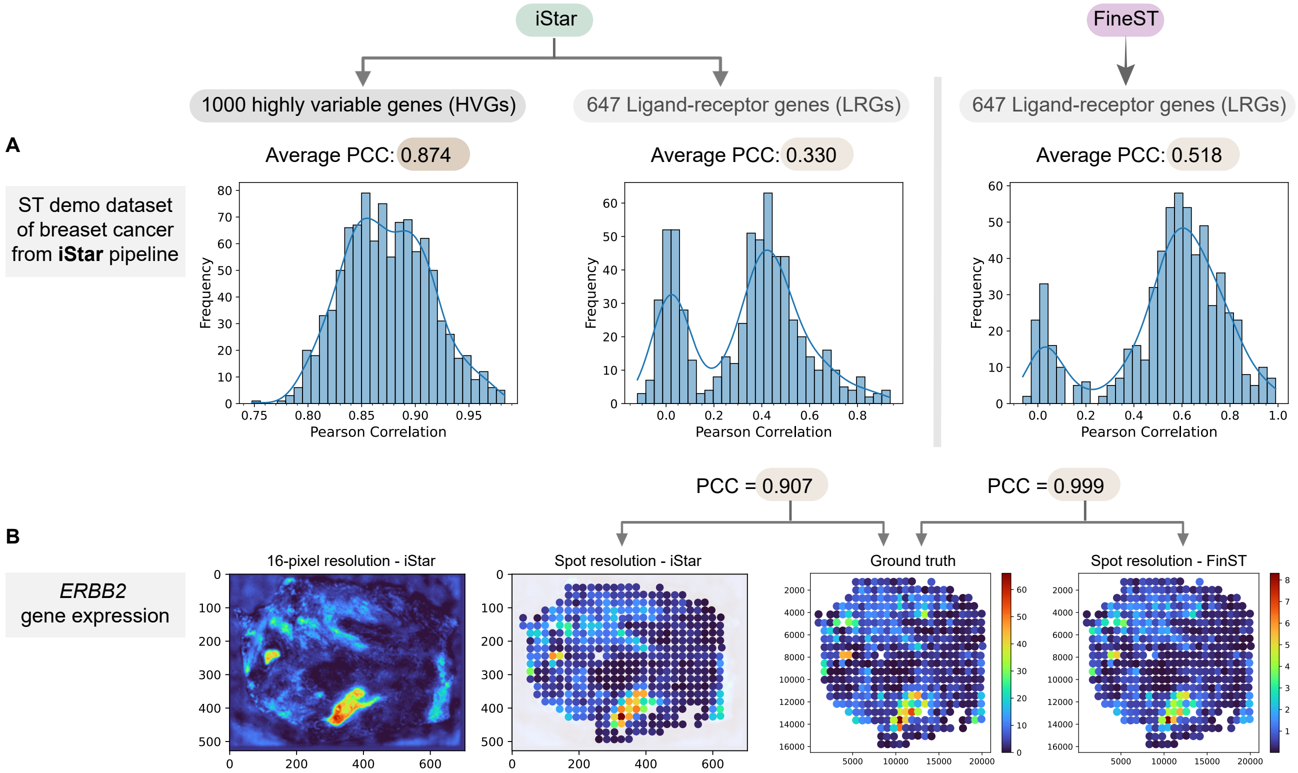

Figure S13. Comparison of inference performance between iStar and FineST methods. (A) On the ST demo dataset of HER2+ breast cancer from iStar pipeline, iStar does achieve good inference on the top 1,000 HVGs (average PCC is 0.974), but worse than FineST on LRGs (average PCC: 0.330 vs 0.518). **(B)** The inferred expression of *ERBB2*, between iStar and FineST, at spot resolution. FineST gets a higher PCC when compared with the ground truth.

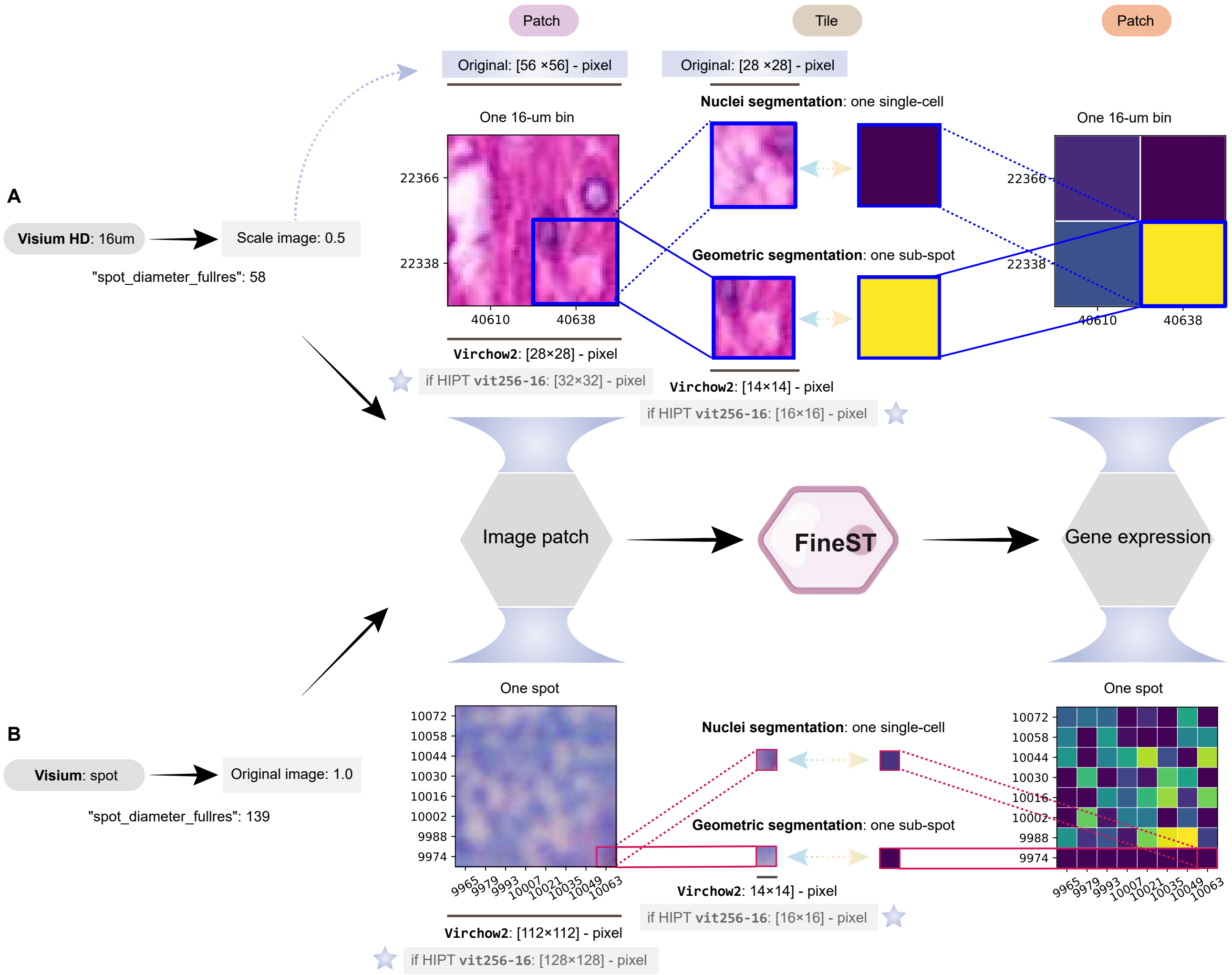

Figure S14. The HE image segmentation for sub-spot and single-nuclei in FineST framework. (A) On VisiumHD data: CRC dataset with 16μm resolution, here the HE image is scaled with scale factor 0.5, thus [28×28]-pixel of the scaled image matches [56×56]-pixel of the original image. [56×56]-pixel is roughly about the value of 'spot_diameter_fullres' of 16μ bin with 58 pixels. **(B)** On Visium data: NPC dataset with spot-resolution, here it directly segments the original image into [112×112]-pixel patches, which is roughly about the value of 'spot_diameter_fullres' of 139 pixels.

Supplementary References

1. Xun, Z. *et al.* Reconstruction of the tumor spatial microenvironment along the malignant-boundary-nonmalignant axis. *Nature Communications* **14**, 933 (2023).

2. Li, Z., Wang, T., Liu, P. & Huang, Y. SpatialDM for rapid identification of spatially co-expressed ligand-receptor and revealing cell-cell communication patterns. *Nature Communications* **14**, 3995 (2023).

3. Oliveira, M. F. d. *et al.* High-definition spatial transcriptomic profiling of immune cell populations in colorectal cancer. *Nature Genetics* 1–12 (2025).

4. Janesick, A. *et al.* High resolution mapping of the tumor microenvironment using integrated single-cell, spatial and in situ analysis. *Nature Communications* **14**, 8353 (2023).

5. Gong, L. *et al.* Nasopharyngeal carcinoma cells promote regulatory T cell development and suppressive activity via CD70-CD27 interaction. *Nature Communications* **14**, 1912 (2023).

6. Andersson, A. *et al.* Spatial deconvolution of HER2-positive breast cancer delineates tumor-associated cell type interactions. *Nature Communications* **12**, 6012 (2021).

7. Berdiel-Acer, M. *et al.* Stromal NRG1 in luminal breast cancer defines pro-fibrotic and migratory cancer-associated fibroblasts. *Oncogene* **40**, 2651–2666 (2021).

8. Marafie, S. K. *et al.* Exploring the binding mechanism of NRG1–ERBB3 complex and discovery of potent natural products to reduce diabetes-assisted breast cancer progression. *Interdisciplinary Sciences: Computational Life Sciences* **15**, 452–464 (2023).

9. Chen, Y., Lu, A., Hu, Z., Li, J. & Lu, J. ERBB3 targeting: A promising approach to overcoming cancer therapeutic resistance. *Cancer Letters* **599**, 217146 (2024).

10. Liu, M. *et al.* INHBA is a mediator of aggressive tumor behavior in HER2+ basal breast cancer. *Breast Cancer Research* **24**, 18 (2022).

11. Du, R. *et al.* Activin receptors in human cancer: Functions, mechanisms, and potential clinical applications. *Biochemical Pharmacology* **222**, 116061 (2024).

12. McCluney, S. *et al.* Anti-activin treatment increases T cell infiltration in breast and pancreatic tumours and promotes survival in a SMAD4-null mouse pancreatic cancer model. *bioRxiv* 2025–06 (2025). URL `https://www.biorxiv.org/content/early/2025/12/23/2025.06.13.659133`.

13. Zimmermann, E. *et al.* Virchow2: Scaling self-supervised mixed magnification models in pathology. *arXiv preprint arXiv:2408.00738* (2024).

14. Chen, R. J. *et al.* Scaling vision transformers to gigapixel images via hierarchical self-supervised learning. In *Proceedings of the IEEE/CVF Conference on Computer Vision and Pattern Recognition*, 16144–16155 (2022).

15. Zhang, D. *et al.* Inferring super-resolution tissue architecture by integrating spatial transcriptomics with histology. *Nature Biotechnology* 1–6 (2024).

16. Hu, J. *et al.* Deciphering tumor ecosystems at super resolution from spatial transcriptomics with TESLA. *Cell Systems* **14**, 404–417 (2023).

17. Ding, Q. *et al.* Dimension reduction, cell clustering, and cell–cell communication inference for single-cell transcriptomics with DcjComm. *Genome Biology* **25**, 241 (2024).

18. Li, B., Wang, C., Wang, Y., Li, P. & Liu, Z.-P. RegNetwork 2025: An integrative data repository for gene regulatory networks in human and mouse. *Nucleic Acids Research* gkaf779 (2025).

19. Erickson, A. *et al.* Spatially resolved clonal copy number alterations in benign and malignant tissue. *Nature* **608**, 360–367 (2022).

20. You, R. *et al.* Single-cell and spatial transcriptomics reveal mechanisms of radioresistance and immune escape in recurrent nasopharyngeal carcinoma. *Nature Genetics* 1–16 (2025).
