# Supplementary material for: FineST: contrastive learning integrates histology and spatial transcriptomics for nuclei-resolved ligand-receptor analysis
Source: Nat Commun. 2026 Mar 16;17:4645. doi: 10.1038/s41467-026-70528-7 (PMC13201544; doi:10.1038/s41467-026-70528-7)
Supplement: Supplementary file 2 — Reporting Summary [file 41467_2026_70528_MOESM2_ESM.pdf]

nature portfolio

Corresponding author(s): Yuanhua Huang

Last updated by author(s): Dec 4, 2025

Reporting Summary

Nature Portfolio wishes to improve the reproducibility of the work that we publish. This form provides structure for consistency and transparency in reporting. For further information on Nature Portfolio policies, see our Editorial Policies and the Editorial Policy Checklist.

Statistics

For all statistical analyses, confirm that the following items are present in the figure legend, table legend, main text, or Methods section.

n/a	Confirmed	
☐	☒	The exact sample size (*n*) for each experimental group/condition, given as a discrete number and unit of measurement
☐	☒	A statement on whether measurements were taken from distinct samples or whether the same sample was measured repeatedly
☐	☒	The statistical test(s) used AND whether they are one- or two-sided<br>*Only common tests should be described solely by name; describe more complex techniques in the Methods section.*
☒	☐	A description of all covariates tested
☐	☒	A description of any assumptions or corrections, such as tests of normality and adjustment for multiple comparisons
☐	☒	A full description of the statistical parameters including central tendency (e.g. means) or other basic estimates (e.g. regression coefficient) AND variation (e.g. standard deviation) or associated estimates of uncertainty (e.g. confidence intervals)
☐	☒	For null hypothesis testing, the test statistic (e.g. *F*, *t*, *r*) with confidence intervals, effect sizes, degrees of freedom and *P* value noted<br>*Give P values as exact values whenever suitable.*
☐	☒	For Bayesian analysis, information on the choice of priors and Markov chain Monte Carlo settings
☒	☐	For hierarchical and complex designs, identification of the appropriate level for tests and full reporting of outcomes
☐	☒	Estimates of effect sizes (e.g. Cohen's *d*, Pearson's *r*), indicating how they were calculated

Our web collection on statistics for biologists contains articles on many of the points above.

Software and code

Policy information about availability of computer code

Data collection	The software for FineST is developed based on Python (v3.8.20) and is available from https://github.com/StatBiomed/FineST, together with main analysis notebooks.<br>The development release used for the paper is available here: https://github.com/StatBiomed/FineST/blob/main/docs/source/release.rst
Data analysis	The data processing and analysis supporting the findings in this study can be reproduced using the scripts and notebooks available at: https://finest-rtd-tutorial.readthedocs.io/en/latest/<br><br>Software packages used for the analysis include:<br>FineST(v0.1.0); numpy(v>1.9.0); scipy(v>1.4.0); statsmodels(v>0.11); pandas(v2.0.3); h5py(v3.10.0); openpyxl(v3.1.5); pyreadr(v0.5.2); scikit-learn(v>0.23); anndata(v0.9.2); scanpy(v1.9.8); SpatialDE; SpatialDM(v0.2.0) SparseAEH(v0.1.0); transpa(v0.1.1); squidpy(v1.2.3); stardist (v0.9.1); tensorflow(v2.13.1); pyarrow(v17.00); pykdtree(v1.3.7); scikit-image. CellChatDB was cached via SpatialDM and is versioned in FineST (v0.1.0)

For manuscripts utilizing custom algorithms or software that are central to the research but not yet described in published literature, software must be made available to editors and reviewers. We strongly encourage code deposition in a community repository (e.g. GitHub). See the Nature Portfolio guidelines for submitting code & software for further information.

Data

Policy information about availability of data

All manuscripts must include a data availability statement. This statement should provide the following information, where applicable:

- Accession codes, unique identifiers, or web links for publicly available datasets
- A description of any restrictions on data availability
- For clinical datasets or third party data, please ensure that the statement adheres to our policy

The VisiumHD data and Chromium Single Cell Gene Expression Flex of human colorectal cancer (CRC) were downloaded from the 10x Genomics datasets here: https://www.10xgenomics.com/products/visium-hd-spatial-gene-expression/dataset-human-crc with `Visium HD, Sample P2 CRC' and `Chromium Single Cell Flex, aggregated' Files.

The raw and processed Visium, Xenium spatial sequencing data, and Chromium Single Cell Gene Expression Flex of human breast cancer (BRCA) tissues were downloaded from 10x Genomics: https://www.10xgenomics.com/products/xenium-in-situ/preview-dataset-human-breast with `Visium Spatial', `In Situ Sample 1, Replicate 1' and `FRP' Files, and GEO under accession numbers: https://www.ncbi.nlm.nih.gov/geo/query/acc.cgi?acc=GSE243280 with `GSM7782699', `GSM7780153' and `GSM7782698' Samples. Cell type annotations for Xenium, Visium, and Chromium Flex datasets of BRCA are available for download in the `Cell Type Annotations' Section.
The raw and processed Visium spatial sequencing data of human hepatocellular carcinoma (HCC) tissues were downloaded from Mendeley Data: datasets/skrx2fz79n/1, while the cell type annotations and high-resolution HE-stained images were provided by the corresponding author.
The raw and processed Visium spatial sequencing data of human primary nasopharyngeal carcinoma (NPC) tissues were downloaded from GEO: https://www.ncbi.nlm.nih.gov/geo/query/acc.cgi?acc=GSE200310. The integrated NPC scRNA-seq data of NPC were obtained from our colleagues and are available from the corresponding author upon request.

For easier reuse, we also included them in the FineST Python package as follows, the CRC data: FineST.datasets.CRC16um(), FineST.datasets.CRC08um(), the BRCA data: FineST.datasets.BRCA(), the HCC data: FineST.datasets.HCCP1T(), FineST.datasets.HCCP7T(), and the NPC data: FineST.datasets.NPC().

The ligand-receptor databases are available from the CellChat repository: https://github.com/sqjin/CellChat/tree/master/data.
All data analyzed in this work are available through the figshare link: https://figshare.com/articles/dataset/FineST_supplementary_data/26763241

Research involving human participants, their data, or biological material

Policy information about studies with human participants or human data. See also policy information about sex, gender (identity/presentation), and sexual orientation and race, ethnicity and racism.

Reporting on sex and gender	N/A
Reporting on race, ethnicity, or other socially relevant groupings	N/A
Population characteristics	N/A
Recruitment	N/A
Ethics oversight	N/A

Note that full information on the approval of the study protocol must also be provided in the manuscript.

Field-specific reporting

Please select the one below that is the best fit for your research. If you are not sure, read the appropriate sections before making your selection.

☒ Life sciences ☐ Behavioural & social sciences ☐ Ecological, evolutionary & environmental sciences

For a reference copy of the document with all sections, see nature.com/documents/nr-reporting-summary-flat.pdf

Life sciences study design

All studies must disclose on these points even when the disclosure is negative.

Sample size

All datasets used in this paper are publicly available and were not generated for this study.

We used four cancer datasets to demonstrate our methods:

First, the CRC dataset was generated with the advanced VisiumHD ST platform, the processed 16um CRC gene expression matrix contains spatial information of 137,051 squares and 18,085 genes, while the original 8um matrix has 545,913 squares.
Second, the BRCA datasets were generated from two ST platforms: Visium (4,992 spots and 18,085 genes) and Xenium (167,780 cells and 313 genes). After registration with Visium, the Xenium was aggregated into 3,996 pseudo-Visium spots for ground truth analysis.
Next, the HCC datasets, which include two conditions (ICB non-responder and responder) from Visium: P1_T: 3,348 spots and 36,601 genes;

	and P7_T: 4,106 spots and 36,601 genes.<br>Finally, the NPC dataset was generated by the 10x Genomics Visium v1 platform, converting 1,331 spots and 36,601 genes.<br><br>The sample size for each study is reported in the 'Datasets collection and transcriptome pre-processing' subsection of the Methods section, and 'Table S1. The datasets analyzed in FineST' in Supplementary Information.
Data exclusions	We only removed features following standard procedures as described in the manuscript. No samples are excluded from analysis.
Replication	This is not relevant for this study as no wet lab experiments were performed. The replication and code for computational experiments are deposited at https://finest-rtd-tutorial.readthedocs.io/en/latest/. All attempts at replication of data analysis were successful. More than five replications for real data have been performed.
Randomization	Randomization usually applies to studies involving participants, cells, or animals. This is not relevant for this study as no wet lab experiments were performed.
Blinding	Similar to the reason above, blinding is not relevant for this study as not relevant to our study, as we report an analysis software as the main finding, and no wet lab experiments were performed.

Reporting for specific materials, systems and methods

We require information from authors about some types of materials, experimental systems and methods used in many studies. Here, indicate whether each material, system or method listed is relevant to your study. If you are not sure if a list item applies to your research, read the appropriate section before selecting a response.

Materials & experimental systems

n/a	Involved in the study
☒	☐ Antibodies
☒	☐ Eukaryotic cell lines
☒	☐ Palaeontology and archaeology
☒	☐ Animals and other organisms
☒	☐ Clinical data
☒	☐ Dual use research of concern
☒	☐ Plants

Methods

n/a	Involved in the study
☒	☐ ChIP-seq
☒	☐ Flow cytometry
☒	☐ MRI-based neuroimaging

Plants

Seed stocks	N/A
Novel plant genotypes	N/A
Authentication	N/A
